# Determinants of poor glycaemic control in type 2 diabetes in Dire Dawa City, Eastern Ethiopia: a case–control study

**DOI:** 10.1093/inthealth/ihaf024

**Published:** 2025-03-25

**Authors:** Alemayehu Molla Tekalign, Hanna Lambero, Aboma Motuma

**Affiliations:** Department of Internal Medicine, School of Medicine, Dire Dawa University, Dire Dawa, Ethiopia; Bilal Hospital, Dire Dawa, Ethiopia; School of Nursing and Midwifery, College of Health and Medical Sciences, Haramaya University, Harar, Ethiopia

**Keywords:** determinants, Ethiopia, HbA1c, poor glycaemic control, type 2 DM

## Abstract

**Background:**

In Ethiopia, there is little evidence on the factors contributing to poor glycaemic control. This study aimed to identify determinants of poor glycaemic control among type 2 diabetes in Dire Dawa City, Eastern Ethiopia.

**Methods:**

A facility-based unmatched case–control study was conducted among type 2 diabetes mellitus patients in follow-up clinics. Cases were people with glycated haemoglobin (HbA1c) ≥7%, while those with HbA1c <7% were controls. A total of 190 patients were recruited in chronic follow-up diabetic clinics. The data were collected using structured questionnaire interviews and extracted from medical charts and entered into EpiData (EpiData Association, Odense, Denmark) and exported to Stata version 16.1 (StataCorp, College Station, TX, USA) for analysis. A binary logistic regression model was fitted to identify the determinants of poor glycaemic control.

**Results:**

The study showed that being female (adjusted odds ratio [AOR] 2.73 [95% confidence interval {CI} 1.10 to 6.79]), a smoker (AOR 14.85 [95% CI 5.25 to 42.88]), blood glucose monitoring ≤3 times per week (AOR 4.87 [95% CI 1.42 to 16.71]), overweight (AOR 4.96 [95% CI 1.82 to 13.52]) or obese (AOR 5.19 [95% CI 1.76 to 15.56]), ≥10 y on treatment (AOR 3.56 [95% CI 1.17 to 10.82]), having coronary artery disease (AOR 2.47 [95% CI 1.01 to 6.03]) and poor adherence to diabetic medication (AOR 0.24 [95% CI 0.10 to 0.63]) were found to be predictors of poor glycaemic control.

**Conclusions:**

Smoking, overweight or obese, poor medication adherence and blood glucose levels lead to poor glycaemic control. This study shows the benefits of quitting smoking, maintaining a healthy weight, adhering to medication and monitoring blood glucose levels.

## Introduction

Globally, type 2 diabetes mellitus (T2DM) affects 9.3% of the world's population,^1,2^ and more than four million deaths occur each year, with half of those deaths occurring before the age of 70 y.^2^ About 422 million people worldwide have T2DM, the majority living in low- and middle-income countries, and 1.5 million deaths are directly attributed to T2DM each year.^3^ Globally, there were 21 669.9 new cases and a total of 437 906.6 cases of T2DM in 2019 per 100 000 population.^3^ According to the World Health Organization (WHO), T2DM will be the seventh leading cause of death in 2030, affecting approximately 62 million people by the end of 2045.^4–6^ Furthermore, the prevalence of T2DM increased from 4.7% to 8.8% between 1980 and 2017.^7^

The challenge in combating T2DM in sub-Saharan Africa (SSA) is a scarcity of funding, a shortage of diagnostic and management protocols and medications and disparities between public and private sector healthcare.^8^ T2DM affects 4 million adults ages 20–79 y in SSA, with a regional prevalence of 3.9%.^9,10^ More than half (55.8%) of T2DM patients in the region live in one of four countries: South Africa (4.6 million), Nigeria (2.7 million), Democratic Republic of Congo (1.8 million) and Ethiopia (1.7 million).^10–12^

Ethiopia is a low-income country with a rapid transition towards a western lifestyle due to demographic and urbanization changes.^9^ This transition accelerates the spread of non-communicable (NCDs) such as diabetic diseases. The economic costs of NCDs are significant and are due principally to their impact on the non-health sector (reduced workforce and productivity). Even though the cause of diabetes is unknown, many dietary behaviours, including saturated fat consumption, a lack of dietary fibre and active smoking have been linked to the risk of diabetes.^10,11^ It has also been linked to risk factors such as poor diet, sedentary behaviour, obesity and dyslipidaemia.^13^ For instance, the pooled prevalence of T2DM in Ethiopia is estimated at 6.5%.^14^ However, a community-based study in Dire Dawa City found that the prevalence of T2DM was 14.9%, with 41.5% being undiagnosed.^15^

The American Diabetes Association's glycated haemoglobin (HbA1c) target of 7% for non-pregnant adults is considered good control, while HbA1c levels ≥8% are considered poor.^16^ One of the most serious health issues in people with T2DM is poor glycaemic control.

The pooled prevalence of good glycaemic control in SSA was 30%, with a range of 10–60%.^17^ Older age, gender, absence of health insurance, low level of education, place of residence, family history of diabetes, longer duration of T2DM, pill burden, treatment regimen, side effects, alcohol consumption, smoking, presence of comorbidities and poor management are associated with poor glycaemic control.^17^ In contrast, positive perceived family support, adequate coping strategies, dietary adherence, exercise practice, attendance to follow-up and medication adherence are associated with good glycaemic control.^18^

In Ethiopia, a pooled meta-analysis showed that poor glycaemic control was 61.1% in people with T2DM,^19^ and in Dire Dawa, poor glycaemic control among T2DM patients was 45.2%.^20^ The majority of the studies in Ethiopia, such as in Jimma,^13^ Gondar,^21^ Dire Dawa,^15,20^ Southern Ethiopia,^22,23^ and Addis Ababa,^18^ used a fasting blood glucose test. However, the International Diabetes Federation recommends that the HbA1c test is a good predictor of glycaemic control for T2DM over a long period.^20^ Despite this, monitoring glycaemic and long-term complications using the HbA1c test was rare in Ethiopia. Therefore, the current study aimed to identify determinants of poor glycaemic control using HbA1c among people with T2DM in chronic follow-up clinics in selected hospitals in Dire Dawa City, Eastern Ethiopia.

## Methods

### Study setting and period

Dire Dawa City is located 515 km east of Addis Ababa, Ethiopia's capital city. Based on Ethiopian fiscal year (EFY) 2012 figures from the Central Statistical Agency of Ethiopia, Dire Dawa City has an estimated total population of 506 639, consisting of 185 377 males and 184 264 females. Of that population, 32.5% are rural inhabitants and 67.5% are urban dwellers.^24,25^ According to the 2015 (EFY) health and health-related indicators published by Ministry of Health, Dire Dawa City has 3 public hospitals, 3 private hospitals, 15 health centres and 36 health posts. The research was carried out in the Bilal Hospital (private) and Dil Chora Referral Hospital (public) from May to June 2022. The total number of patients seen at Dile Chora Referral Hospital was 200 000. It has 300 beds, 353 administrative staff, 62 doctors and 104 nurses. At Bilal Hospital, 93 000 patients were seen. It has 92 beds, 30 doctors and 80 nurses. These two hospitals were selected because they have well-established chronic disease follow-up clinics for T2DM and cardiovascular disease. Both hospitals are the largest in the area, offering comprehensive multispecialty care in a variety of disciplines. Patients served in the hospitals come from Dire Dawa City and surrounding regional states, Oromia, Somalia, Somaliland and Djibouti.

### Study design and population

An unmatched case–control study was conducted among people with T2DM. People with poor glycaemic control were cases and those with good glycaemic control were controls. The study participants were selected from diabetes follow-up clinics during the data collection period and met the eligibility criteria. Adults (≥18 y of age) with diabetes who had at least three consecutive follow-ups and who had at least one HbAlc test were eligible for both cases and controls. However, pregnant women and those who were mentally unstable or critically ill and unable to respond were excluded from the study.

### Sample size and sampling technique

The sample size was calculated using OpenEpi version 3 (www.openepi.com) by taking two population proportions and assuming a 95% significance level, 80% power and a case:control ratio of 1:1. The odds ratio (OR) and proportion of different predictor variables of glycaemic control among cases and controls were derived from a study conducted in Jimma, Ethiopia.^13^ The largest sample size was considered (n=190), with 95 cases and 95 controls. The study participants were people with T2DM who had follow-ups at Dil Chora Referral Hospital and Bilal Hospital. There were 850 people with T2DM follow-up appointments at the Dil Chora Referral Hospital outpatient follow-up clinic and 156 people with T2DM follow-up appointments at Bilal Hospital during the study period.^26^ First, we developed a sample frame of patients who had an HbA1c test in either hospital. These two hospitals were selected purposively because they have separate clinics for diabetes follow-up and HbA1c tests.

The number of study participants was determined from the total number of people with diabetes who were followed up in the two hospitals (n=1006). Samples were allocated to each selected hospital based on the proportion of the study participants. The lists of sampling frames were obtained from the updated registration books of the follow-up clinics. After establishing the sampling frames of respondents, a simple random sampling technique was used to identify the study unit to be included in the study.

### Data collection instruments and procedures

The questionnaires were developed after reviewing previously published literature.^19,20^ Face-to-face interviews were conducted using a structured questionnaire to collect sociodemographic information such as gender, age, marital status, education status, occupation, residence, religion, clinical and behavioural characteristics, duration of diabetes, family history of diabetes, lifestyle and self-care (drug adherence, smoking, physical activity). Following completion of the interview, using the data abstraction format information on comorbid disease, diabetic complications, weight, height (body mass index [BMI]), type of drug treatment, recent HbA1c, systolic blood pressure, diastolic blood pressure, lipid profiles, serum creatinine value and urine dipstick semi-qualitative protein level, chronic complications of diabetes were abstracted from the participants’ medical records.

### Variables and measurement

The HbA1c test was used to measure the outcome variable. HbA1c levels were categorized into two groups based on the American Diabetes Association and International Diabetes Federation guidelines:^27,28^ good glycaemic control if the HbA1c level is <7% and poor glycaemic control when it is ≥7%. Sociodemographic characteristics (age, gender, education status, marital status and occupation) were determined in face-to-face interviews.

BMI was categorized according to WHO criteria (underweight: BMI <18.5 kg/m^2^; normal: BMI 18.5–24.9 kg/m^2^; overweight: BMI 25.0–29.9 kg/m^2^; and obese: BMI ≥30.0 kg/m^2^). Smoking status was categorized as never smoked and lifetime smoker. In this study we asked about the drinking habits of the participants. The alcohol drinking habits of the study participants during the survey period were classified as never, occasional and frequent drinker. The total physical activity score was computed as the sum of all metabolic equivalent (MET) minutes/week for vigorous-intensity physical activity, moderate-intensity physical activity and walking. The sum of MET minutes/week was categorized as high (≥3000 MET minutes), recorded as ‘high physical activity’ or ‘yes’, and low (<2999 MET minutes), recorded as ‘low physical activity’ or ‘no’.^29^ Biochemical markers were determined, including serum creatinine (normal range 0.7–1.3 mg/dl [61.9–114.9 µmol/l] for men and 0.6–1.1 mg/dl [53–97.2 µmol/l] for women) and the blood lipid profile (normal values: low-density lipoprotein [LDL] cholesterol <100 mg/dl, high-density lipoprotein [HDL] cholesterol >40 mg/dl, triglycerides <150 mg/dl and total cholesterol <200 mg/dl).^29^ A healthy diet that is naturally rich in nutrients and low in fat and calories is fruits, vegetables and whole grains three or more times per week.^30^ Diabetic complications, clinically related factors (duration of diabetes and comorbidities), blood pressure measurement and drug regimens were also extracted from the medical records.

### Data quality assurance

Six nurses were recruited for data collection who work in the chronic disease follow-up clinics of the study hospitals. Training was provided for 2 d by the principal investigator on the data collection process and focused on the content of the questionnaire, data collection techniques, ethical human research and how to enter the data provided. Before the data collection, a pretest was conducted with 5% of the sample size in a similar setting at Hiwot Fana Specialized Comprehensive University Hospital in Harar, and amendments were made based on the pretest results. The principal investigator oversaw and checked on the completeness and consistency of the questionnaires.

### Data processing and analysis

Data were collected and entered to EpiData version 3.1 (EpiData Association, Odense, Denmark) before being exported to the Stata 16.1 (StataCorp, College Station, TX, USA) for analysis. The data were checked for outliers, missing values and assumptions. People with T2DM with an HbA1c ≥7% were classified as cases and those with values < 7% were controls. Descriptive results were presented using frequency, proportion, and mean. In bivariable logistic regression analysis, p-values <0.25 were chosen as candidates for multivariable logistic regression analysis. The multicollinearity was checked. Model fitness was tested with the Hosmer–Lemeshow goodness-of-fit test with a p-value of 0.7654. Finally, a multivariable logistic regression analysis was done to identify predictors of poor glycaemic control. The adjusted odds ratio (AOR) with 95% confidence interval (CI) was reported to show the degree of association between the independent and the outcome variable at p<0.05.

## Results

### Sociodemographic characteristics of the study participants

A total of 190 study participants (95 cases and 95 controls) were included in the study. The mean age of the cases was 56±13.75 y and that of the control group was 53±12.23 y. The mean age difference between cases and controls was not statistically significant (p=0.245). More than three-quarters of cases and controls were ≥45 y of age. A total of 55% of cases and 44.6% of controls were married. Most of the study participants, 42.9% of cases and 57.1% of controls, had no formal education. Almost half of the study participants were unemployed and 15.8% of T2DM patients live >10 y with a mean HbA1c test of 13% (Table 1).

**Table 1.** Sociodemographic characteristics of adults with T2DM at chronic follow-up in selected hospitals in Dire Dawa, Eastern Ethiopia, 2022

Variables	Cases, n (%)	Controls, n (%)	Total, n (%)
Sex			
Male	30 (43.5)	39 (56.5)	69 (36.3)
Female	65 (53.7)	56 (46.3)	121 (63.7)
Age (completed years)			
25–34	4 (66.7)	2 (33.3)	6 (3.2)
35–44	19 (52.80)	17 (47.2)	36 (19.0)
45–54	22 (46.8)	25 (53.2)	47 (24.7)
55–64	28 (56.0)	22 (44.0)	50 (26.3)
≥65	22 (43.1)	29 (56.9)	51 (26.8)
Marital status			
Unmarried	6 (46.2)	7 (53.8)	13 (6.8)
Married	72 (55.4)	58 (44.6)	130 (68.5)
Widowed	11 (32.4)	23 (67.6)	34 (17.9)
Divorced	6 (46.2)	7 (53.9)	13 (6.8)
Education status			
No formal education	33 (42.9)	44 (57.1)	77 (40.5)
Primary education	25 (55.6)	20 (44.4)	45 (23.7)
Secondary education	27 (58.7)	19 (41.3)	46 (24.2)
College and above	10 (45.5)	12 (54.5)	22 (11.6)
Occupation			
Employed	45 (50.0)	45 (50.0)	90 (47.4)
Unemployed	50 (50.0)	50 (50.0)	100 (52.6)
Duration of diabetes (years)			
<5	55 (52.4)	50 (47.6)	105 (55.1)
5–10	25 (45.4)	30 (54.6)	55 (29.1)
>10	15 (50.0)	15 (50.0)	30 (15.8)

### Behavioural and clinical characteristics of the study participants

Of the 190 participants interviewed, 19 (20%) cases and 9 (9.5%) controls monitored their blood glucose three or more times per week. More than half of the cases (54 [56.8%]) and 10 (10.5%) of the controls were cigarette smokers. A total of 19 (20%) of the cases and 9 (9.5%) of the controls had alcohol consumption habits. The study showed that 37.9% of cases and 25.3% of controls were overweight and 34.7% of cases and 14.7% of controls were obese. A total of 61 (64.2%) cases and 43 (45.3%) controls were hypertensive. Regarding adherence to diabetic medication, 42 (44.2%) cases and 15 (15.8%) controls had no adherence to their diabetic medication. A total of 68.4% of cases and 82.1% of controls consume a healthy diet less than three times per week (including fruits, vegetables, high fibre and low unsaturated fat). Regarding physical activity, 83.2% of cases and 84.2% of controls engaged in physical activity. A total of 61 (58.7%) cases and 43 (41.3%) controls had high blood pressure during data collection or were already diagnosed as hypertensive. Of the study participants, 36 (37.9%) of the cases and 13 (13.7%) of the controls had thyroid dysfunction (Table 2).

**Table 2.** Behavioural characteristics and clinical information of people with T2DM at Dil Chora and Bilal hospitals in the chronic diabetics clinic, Dire Dawa City, Eastern Ethiopia, 2022 (N=190)

Variables	Categories	Cases, n (%)	Controls, n (%)	p-Value
Physical activity	Yes	79 (83.2)	80 (84.2)	0.844
	No	16 (16.8)	15 (15.8)	
Blood glucose monitoring	≥3 times/week	19 (20.0)	9 (9.5)	0.041
	<3 times/week	76 (80.0)	86 (90.5)	
Lifetime smoking	Yes	54 (56.8)	10 (10.5)	0.001
	No	41 (43.2)	85 (89.5)	
Alcohol consumption	Occupational/frequent	19 (20.0)	9 (9.5)	0.041
	Not at all	76 (80.0)	86 (90.5)	
Hypertension	Yes	61 (64.2)	43 (45.3)	0.009
	No	34 (35.8)	52 (54.7)	
Frequency of healthy diet	≥3 times/week	30 (31.6)	17 (17.9)	0.029
	<3 times/week	65 (68.4)	8 (82.1)	
Thyroid dysfunction	Yes	37 (38.0)	27 (28.4)	0.125
	No	58 (61.0)	68 (71.6)	
Healthy diet	Yes	37 (38.9)	22 (23.2)	0.019
	No	58 (61.1)	73 (76.8)	
Adherence to diabetic medications	Adhered	42 (44.2)	15 (15.8)	0.001
	Non-adhered	53 (55.8)	80 (84.2)	
BMI (kg/m^2^)	<25	26 (27.4)	57 (60.0)	0.001
	25–29.9	36 (37.9)	24 (25.3)	
	≥30	33 (34.7)	14 (14.7)	

### Biochemical and anthropometric characteristics of the study participants

The mean cholesterol was 202.02±58.8 mg/dl in cases and 179.24±51.3 mg/dl in controls (p=0.009). Also, the mean triglyceride was high in the cases (175.04±95.98 mg/dl) and controls (149.61±62.27 mg/dl; p=0.005). Moreover, the mean BMI was 28.41±6.33 kg/m^2^ in cases and 24.71±3.71 kg/m^2^ in controls (p<0.001) (Table 3).

**Table 3.** Biochemical and anthropometric characteristics among adults with T2DM in Dil Chora and Bilal hospitals, Dire Dawa City, Eastern Ethiopia, 2022 (N=190)

Clinical variables	HbA1c	Mean	Standard deviation	t-Test	p-Value
Total cholesterol	≥7% (case)	202.24 mg/dl	58.78	−2.36	0.019
	<7% (control)	179.24 mg/dl	51.30		
Triglyceride	≥7% (case)	175.04 mg/dl	95.98	−2.45	0.005
	<7% (control)	149.61 mg/dl	62.27		
HDL	≥7% (case)	46.09 mg/ dl	43.52	1.426	0.156
	<7% (control)	54.69 mg /dl	57.40		
LDL	≥7% (case)	116.84 mg/dl	47.66	−2.47	0.014
	<7% (control)	100.53 mg/dl	43.28		
BMI	≥7% (case)	28.41 kg/m^2^	6.33	−.4.49	0.000
	<7% (control)	24.71 kg/m^2^	3.71		

### Diabetic complications

Regarding diabetic complications, 51 (53.7%) of the cases and 29 (30.5%) of the controls had diabetic neuropathy and 8 (8.4%) of the cases and 7 (7.4%) of the controls had diabetic retinopathy. In addition, 17 (17.9%) cases and 10 (10.5%) controls had nephropathy. Moreover, 48 (50.5%) of the cases had coronary artery disease, while 32 (33.2%) of the controls had coronary artery disease (p<0.05) (Table 4).

**Table 4.** Diabetic complications among adults with T2DM follow-up in Dil Chora and Bilal hospitals, Dire Dawa City, Eastern Ethiopia, 2022 (N=190)

Diabetic complication	Category	Cases, n (%)	Controls, n (%)	p-Value
Diabetic neuropathy	Yes	51 (53.7)	29 (30.5)	0.788
	No	44 (46.3)	66 (69.4)	
Diabetic retinopathy	Yes	8 (8.4)	7 (7.4)	0.001
	No	87 (91.6)	88 (92.6	
Nephropathy	Yes	17 (17.9)	10 (10.5)	0.319
	No	78 (82.1)	85 (89.5)	
Coronary artery disease	Yes	48(50.5%)	32 (33.7)	0.019
	No	47(49.5%)	63 (66.3%)	

### Prescribed medications

The study shows that 65 (68.4%) of the cases and 64 (67.4%) of the controls had been prescribed metformin and 16 (16.8%) of the cases and 7 (7.4%) of the controls had been prescribed insulin. Most of the study participants, 51 (53.7%) of the cases and 60 (63.2%) of the controls, had been prescribed the lipid-lowering agent statin. Angiotensin-converting enzyme inhibitor or angiotensin II receptor blocker had been prescribed for 22 (23.2%) cases and 18 (18.9%) controls with T2DM.

### Predictors of poor glycaemic control

In bivariable logistic regression analysis, adherence to diabetes medication, blood glucose monitoring, smoking, alcohol consumption, hypertension, diabetes sensory neuropathy, coronary artery disease, high BMI and duration of diabetes were found to be associated with poor glycaemic control. However, in the multivariable logistic regression analysis, being female, smoking, adherence to diabetic medication, self-monitoring of blood glucose, coronary artery disease, high BMI and duration of diabetes were significantly associated with poor glycaemic control.

The study shows that females are 2.7 times more likely to have poor glycaemic control compared with men (AOR 2.73 [95% CI 1.10 to 6.79]). Study participants who were practicing self-monitoring of blood glucose less than three times per week were 4.9 times more likely to have poor glycaemic control (AOR 4.87 [95% CI 1.42 to 16.71]) than their counterparts. Furthermore, being a smoker (AOR 14.85 [95% CI 5.21 to 42.28]), having coronary artery disease (AOR 2.4 [95% CI 1.01 to 6.03]), being overweight (AOR 4.69 [95% CI 1.82 to 13.52]) or obese (AOR 5.19 [95% CI 1.76 to 15.16]) and long duration of diabetes (AOR 3.56 [95% CI 1.17 to 10.82]) were significantly associated with poor glycaemic control. In contrast, study participants who adhered to their diabetes medication ≥5 d/week had lower blood glycaemic levels by 76% (AOR 0.24 [95% CI 0.10 to 0.63]) compared with those with adhered <5 d/week (Table 5).

**Table 5.** Determinants of poor glycaemic control people with T2DM in a follow-up clinic in Dil Chora and Bilal hospitals, Dire Dawa City, Eastern Ethiopia, 2022 (N=190)

Variables	Cases	Controls	Crude OR (95% CI)	AOR (95% CI)
Sex				
Male	30	39	1	1
Female	65	56	1.50 (0.83 to 2.74)	**2.73 (1.10 to 6.79)** *
Adherence to diabetic medications				
<5 d/week	42	15	1	1
≥5 d/week	53	80	0.24 (0.12 to 0.47)	**0.24 (0.10 to 0.63)** *
Blood glucose monitoring				
<3 times/week	70	86	2.39 (1.02 to 5.59)	**4.87 (1.42–16.71)** *
≥3 times/week	19	9	1	1
Smoking				
Yes	10	54	11.19 (5.18 to 24.20)	**14.85 (5.21 to 42.28)** *
No	85	41	1	1
Alcohol consumption				
Yes	89	76	3.71 (1.41 to 9.76)	1.41 (0.37 to 5.37)
No	6	19	1	1
Hypertension				
Yes	61	43	2.17 (1.21 to 3.88)	0.51 (0.22 to 1.21)
No	34	52	1	1
Diabetes sensory neuropathy				
Yes	16	10	2.63 (1.46 to 4.78)	0.50 (0.21 to 1.19)
No	78	75	1	1
Coronary artery disease				
Yes	48	32	2.01 (1.12 to 3.61)	2.47(1.01 to 6.03)*
No	47	63	1	1
Total cholesterol (mg/dl)				
<200	46	59	1	1
≥200	49	36	1.75 (0.98 to 3.11)	1.19 (0.43 to 3.25)
Triglyceride (mg/dl)				
<150	42	52	1	1
≥150	53	43	1.53 (0.86 to 2.70)	1.67 (0.73 to 3.82)
HDL (mg/dl)				
≥40	67	57	1	1
<40	28	38	0.63 (0.34 to 1.15)	0.86 (0.34 to 2.19)
LDL (mg/dl)				
<100	34	42	1	1
≥100	61	53	1.42 (0.79 to 2.55)	2.50 (0.54 to 4.14)
BMI (kg/m^2^)				
<25	26	57	1	1
25–29.9	36	24	3.29 (1.64 to 6.58)	4.96 (1.82 to 13.52)*
≥30	33	14	5.17 (2.37 to 11.26)	**5.19 (1.76 to 15.16)** *
Duration of diabetes (years)				
<10	64	87	1	1
≥10	31	8	5.27 (2.27 to 12.22)	**3.56 (1.17 to 10.82)** *

* p<0.05.

## Discussion

This study used a facilities-based unmatched case–control design to determine predictors of glycaemic control using the Hb1Ac test. The study showed that being female, smoking, non-adherence to diabetic medications, having low self-monitoring of blood glucose, having coronary artery disease, being overweight or obese and having a long duration of diabetes were significantly associated with poor glycaemic control.

The ≥60-y age group had a higher proportion of poor glycaemic control, which is in line with a study conducted in Dar es Salaam.^31^ The study showed that 69% of participants were on oral metformin alone (with no insulin as a component of their drug therapy). This finding was consistent with findings reported by Weng et al.^32^

This study revealed that being female was significantly associated with poor glycaemic control supported by a study conducted in India,^33^ Tanzania^31^ and Ethiopia.^34^ This might be because women are responsible for family care and may neglect their healthcare.^33,35^ Hence, women tend to be more obese and have higher bad cholesterol than men with diabetes.^34^ Women in Ethiopia might not attend their follow-up therapy as needed due to their workload at home and are less likely to follow their drug therapy attentively^23,34^ and are less adherent to lifestyle modifications such as regular physical activity.^34,36,37^ A previous study by Motuma et al.^36^ found that more physical inactivity and obesity were observed in Ethiopian women compared with men, as many women in sub-Saharan countries have poor access to treatment, care and education.^17^ Also, women have more adverse effects on lipid profiles than men due to oestrogen effects.^31,36^ This may have a greater role in disturbing insulin action in women and inflammatory factors may interact with female sex hormones.^33^

Being overweight or obese was significantly associated with poor glycaemic control, and this study was consistent with previous reports.^31,32,38^ This might be due to the exacerbation of insulin resistance due to increased fat mass and visceral adiposity, which affect insulin sensitivity and cause insulin resistance.^34^

In line with a study conducted by Demoz et al.,^34^ poor medication adherence was also found to be an important predictor of poor glycaemic control. Non-adherence to diabetic medication was associated with increased levels of HbA1c,^31^ consistent with other reports in Jordan and Ethiopia.^39,40^ Similarly, studies have shown that people with inadequate knowledge of their status might not regularly attend clinics and are less likely to adhere to their medication.^41^ A lack of education affects patients’ motivation to cultivate healthy lifestyles and maintain treatment adherence.^42,43^ However, medication adherence should not be supposed as the patient's only problem.^13,34^ Poor medication adherence might due to failure to agree on the prescription and/or failure to provide support to the patient once the drug has been dispensed.

In the current study, a higher proportion of poor glycaemic control was reported in study participants who lived with T2DM for a long period of time. The result is consistent with previous findings in the southern Sahara and Dil Chora Hospital.^20,44,45^ However, a study conducted at the Gondar University Hospital revealed a high proportion of poor glycaemic control in patients with diabetes for <7 y.^46^ This is possibly due to reduced insulin secretion or excessive insulin resistance in those patients.^33^

In this study, not self-monitoring of blood glucose was a predictor of poor glycaemic control. This finding is consistent with studies in Jordan^39^ and at Jimma University.^13^ In contrast, a study conducted in Mekelle, Ethiopia found that patients who were adherent to self-monitoring of blood glucose had good glycaemic control.^47^ A patient who does not self-monitor their blood glucose may not visit health facilities or consult healthcare providers or adjust their diabetic medication and modify their lifestyle, contributing to poor glycaemic control.

In the current study, participants who had coronary artery disease were found to have poor glycaemic control compared with those who do not have coronary artery disease. Our results are consistent with previous studies on the relation of T2DM and endothelial dysfunction.^48^ Poor glycaemic control exerts an adverse effect on endothelial function and aggravates coronary atherosclerosis in T2DM. These observations provide insights into the management of T2DM patients with coronary artery disease.

### Strengths and limitations

The strength of the study is the unmatched case–control design that includes private and public hospitals and used the HbA1c test, which is a good predictor of glycaemic control. Limitations included recall and social desirability bias. Even though weight and height measurements were taken by health professionals, a single measurement may not be accurate, introducing measurement bias. The sample size was disproportionate, as men were underrepresented. Subjects were T2DM patients who were on follow-up at the outpatient clinic, which may not be representative of the overall diabetes population. Data taken from the medical records may have affected the study results and some factors were not controlled, such as the experience of the managing clinician, types of medication used and other comorbidities.

## Conclusions

According to this study, being female, smoking, self-monitoring blood glucose three or fewer times per week, coronary artery disease, a longer duration of DM (≥10 y) and being overweight or obese were independent predictors of poor glycaemic control. Healthcare providers should encourage people with T2DM to adjust their diabetic medication and modify their lifestyle and take their diabetes medication regularly.

## Data Availability

The dataset is available from the corresponding author upon reasonable request.

## References

[1] International Diabetes Federation . IDF diabetes atlas, ninth edition. Brussels: International Diabetes Federation; 2019.

[2] Arokiasamy P, Salvi S, Selvamani Y. Global burden of diabetes mellitus. In: Haring R, Kickbusch I, Ganten D et al., editors. Handbook of global health. Cham: Springer; 2021:495–538.

[3] Ye J, Wu Y, Yang S et al. The global, regional and national burden of type 2 diabetes mellitus in the past, present and future: a systematic analysis of the Global Burden of Disease Study 2019. Front Endocrinol (Lausanne). 2023;14:1192629. 37522116 10.3389/fendo.2023.1192629 PMC10376703

[4] Saeedi P, Petersohn I, Salpea P et al. Global and regional diabetes prevalence estimates for 2019 and projections for 2030 and 2045: results from the International Diabetes Federation Diabetes Atlas, 9^th^ edition. Diabetes Res Clin Pract. 2019;157:107843. 31518657 10.1016/j.diabres.2019.107843

[5] Cho NH, Shaw J, Karuranga S et al. IDF Diabetes Atlas: global estimates of diabetes prevalence for 2017 and projections for 2045. Diabetes Res Clin Pract. 2018;138:271–81. 29496507 10.1016/j.diabres.2018.02.023

[6] World Health Organization . WHO Diabetes Country Profiles 2016. Profiles DC.’. Geneva: World Health Organization; 2016.

[7] World Health Organization . Global report on diabetes. Geneva: World Health Organization; 2016.

[8] Atun R, Gale EA. The challenge of diabetes in sub-Saharan Africa. Lancet Diabetes Endocrinol. 2015;3(9):675–7. 26201978 10.1016/S2213-8587(15)00236-3

[9] Mbanya JCN, Motala AA, Sobngwi E et al. Diabetes in sub-Saharan Africa. Lancet. 2010;375(9733):2254–66. 20609971 10.1016/S0140-6736(10)60550-8

[10] Ojuka EO, Goyaram V. Increasing prevalence of type 2 diabetes in sub-Saharan Africa: not only a case of inadequate physical activity. Med Sport Sci. 2014;60:27–35. 25226798 10.1159/000357333

[11] Hall V, Thomsen RW, Henriksen O et al. Diabetes in Sub Saharan Africa 1999–2011: epidemiology and public health implications. A systematic review. BMC Public Health. 2011;11:564. 21756350 10.1186/1471-2458-11-564 PMC3156766

[12] Azevedo M, Alla S. Diabetes in sub-Saharan Africa: Kenya, Mali, Mozambique, Nigeria, South Africa and Zambia. Int J Diabetes Dev Ctries. 2008;28(4):101–8. 20165596 10.4103/0973-3930.45268 PMC2822152

[13] Mamo Y, Bekele F, Nigussie T et al. Determinants of poor glycemic control among adult patients with type 2 diabetes mellitus in Jimma University Medical Center, Jimma zone, south west Ethiopia: a case control study. BMC Endocr Disord. 2019;19:91. 31464602 10.1186/s12902-019-0421-0 PMC6716911

[14] Zeru MA, Tesfa E, Mitiku AA et al. Prevalence and risk factors of type-2 diabetes mellitus in Ethiopia: systematic review and meta-analysis. Sci Rep. 2021;11(1):21733. 34741064 10.1038/s41598-021-01256-9 PMC8571297

[15] Ayele BH, Roba HS, Beyene AS et al. Prevalent, uncontrolled, and undiagnosed diabetes mellitus among urban adults in Dire Dawa, Eastern Ethiopia: a population-based cross-sectional study. SAGE Open Med. 2020;8:2050312120975235. 33282310 10.1177/2050312120975235 PMC7686592

[16] Marathe PH, Gao HX, Close KL. American Diabetes Association Standards of Medical Care in Diabetes 2017. J Diabetes. 2017;9(4):320–4. 28070960 10.1111/1753-0407.12524

[17] Fina Lubaki J-P, Omole OB, Francis JM. Glycaemic control among type 2 diabetes patients in sub-Saharan Africa from 2012 to 2022: a systematic review and meta-analysis. Diabetol Metab Syndr. 2022;14(1):134. 36127712 10.1186/s13098-022-00902-0 PMC9487067

[18] Sahile AT, Bekele GE. Prevalence of diabetes mellitus and associated factors in Addis Ababa public health facilities, Addis Ababa, Ethiopia, 2016. Diabetes Metab Syndr Obes. 2020;13:501–8. 32158245 10.2147/DMSO.S237995 PMC7049281

[19] Tegegne KD, Gebeyehu NA, Yirdaw LT et al. Determinants of poor glycemic control among type 2 diabetes in Ethiopia: a systematic review and meta-analysis. Front Public Health. 2024;12:1256024. 38375333 10.3389/fpubh.2024.1256024 PMC10876054

[20] Nigussie S, Birhan N, Amare F et al. Rate of glycemic control and associated factors among type two diabetes mellitus patients in Ethiopia: a cross sectional study. PLoS One. 2021;16(5):e0251506. 33974654 10.1371/journal.pone.0251506 PMC8112661

[21] Legese GL, Asres G, Alemu S et al. Determinants of poor glycemic control among type 2 diabetes mellitus patients at University of Gondar Comprehensive Specialized Hospital, Northwest Ethiopia: unmatched case-control study. Front Endocrinol. 2023;14:1087437. 10.3389/fendo.2023.1087437 PMC9947343 36843610

[22] Zekewos A, Loha E, Egeno T et al. Prevalence of diabetes mellitus and associated factors in southern Ethiopia: a community based study. Ethiop J Health Sci. 2018;28(4):451–60. 30607058 10.4314/ejhs.v28i4.11 PMC6308729

[23] Dawite F, Girma M, Shibiru T et al. Factors associated with poor glycemic control among adult patients with type 2 diabetes mellitus in Gamo and Gofa zone public hospitals, southern Ethiopia: a case–control study. PLoS One. 2023;18(3):e0276678. 36897872 10.1371/journal.pone.0276678 PMC10004482

[24] Tusa BS, Weldesenbet AB, Kebede SA. Spatial distribution and associated factors of underweight in Ethiopia: an analysis of Ethiopian demographic and health survey, 2016. PLoS One. 2020;15(12):e0242744. 33259562 10.1371/journal.pone.0242744 PMC7707465

[25] Central Statistical Agency (CSA)[Ethiopia], ICF . Ethiopia Demographic and Health Survey 2016. Addis Ababa: Central Statistical Agency; 2016.

[26] Amera TG, Tefera YM, Menberu T et al. Determinants of type 2 diabetes mellitus among adults in Dill-Chora Referral Hospital, Dire Dawa, East Ethiopia. Diabetes Metab Syndr Obes. 2022;15:3565–76. 36419502 10.2147/DMSO.S384737 PMC9677892

[27] Ogurtsova K, Guariguata L, Barengo NC et al. IDF Diabetes Atlas: global estimates of undiagnosed diabetes in adults for 2021. Diabetes Res Clin Pract. 2022;183:109118. 34883189 10.1016/j.diabres.2021.109118

[28] Hammack DC . American debates on the legitimacy of foundations. In: Prewitt K, Dogan M, Heydemann S et al., editors. The legitimacy of philanthropic foundations: United States and European perspectives. New York: Russell Sage Foundation; 2006:49–98.

[29] World Health Organization . WHO STEPS surveillance manual: the WHO STEPwise approach to chronic disease risk factor surveillance/noncommunicable diseases and mental health. Geneva: World Health Organization; 2005.

[30] Sami W, Ansari T, Butt NS et al. Effect of diet on type 2 diabetes mellitus: a review. Int J Health Sci (Qassim). 2017;11(2):65–71. PMC5426415 28539866

[31] Kamuhabwa AR, Charles E. Predictors of poor glycemic control in type 2 diabetic patients attending public hospitals in Dar es Salaam. Drug Healthc Patient Saf. 2014;6:155–65. 25368533 10.2147/DHPS.S68786 PMC4216043

[32] Weng W, Tian Y, Kimball ES et al. Treatment patterns and clinical characteristics of patients with type 2 diabetes mellitus according to body mass index: findings from an electronic medical records database. BMJ Open Diabetes Res Care. 2017;5(1):e000382. 10.1136/bmjdrc-2016-000382 PMC5530246 28761654

[33] Haghighatpanah M, Nejad ASM, Haghighatpanah M et al. Factors that correlate with poor glycemic control in type 2 diabetes mellitus patients with complications. Osong Public Health Res Perspect. 2018;9(4):167–74. 30159222 10.24171/j.phrp.2018.9.4.05 PMC6110332

[34] Demoz GT, Gebremariam A, Yifter H et al. Predictors of poor glycemic control among patients with type 2 diabetes on follow-up care at a tertiary healthcare setting in Ethiopia. BMC Res Notes. 2019;12:207. 30947749 10.1186/s13104-019-4248-6 PMC6449968

[35] Kautzky-Willer A, Kosi L, Lin J et al. Gender-based differences in glycaemic control and hypoglycaemia prevalence in patients with type 2 diabetes: results from patient-level pooled data of six randomized controlled trials. Diabetes Obes Metab. 2015;17(6):533–40. 25678212 10.1111/dom.12449 PMC6680342

[36] Motuma A, Gobena T, Roba KT et al. Metabolic syndrome among working adults in eastern Ethiopia. Diabetes Metab Syndr Obes. 2020;13:4941–51. 33363392 10.2147/DMSO.S283270 PMC7753886

[37] Motuma A, Gobena T, Roba KT et al. Sedentary behavior and associated factors among working adults in eastern Ethiopia. Front Public Health. 2021;9:693167. 10.3389/fpubh.2021.693176 PMC8452899 34557467

[38] Sendekie AK, Teshale AB, Tefera YG. Glycemic control in newly insulin-initiated patients with type 2 diabetes mellitus: a retrospective follow-up study at a university hospital in Ethiopia. PLoS One. 2022;17(5):e0268639. 35617250 10.1371/journal.pone.0268639 PMC9135271

[39] Khattab M, Khader YS, Al-Khawaldeh A et al. Factors associated with poor glycemic control among patients with type 2 diabetes. J Diabetes Complications. 2010;24(2):84–9. 19282203 10.1016/j.jdiacomp.2008.12.008

[40] Abebe SM, Berhane Y, Worku A et al. Level of sustained glycemic control and associated factors among patients with diabetes mellitus in Ethiopia: a hospital-based cross-sectional study. Diabetes Metab Syndr Obes. 2015;8:65–71. 25657591 10.2147/DMSO.S75467 PMC4315535

[41] Miras AD, Risstad H, Baqai N et al. Application of the International Diabetes Federation and American Diabetes Association criteria in the assessment of metabolic control after bariatric surgery. Diabetes Obes Metab. 2014;16(1):86–9. 23841525 10.1111/dom.12177

[42] Raum E, Krämer HU, Rüter G et al. Medication non-adherence and poor glycaemic control in patients with type 2 diabetes mellitus. Diabetes Res Clin Pract. 2012;97(3):377–84. 22763108 10.1016/j.diabres.2012.05.026

[43] Letta S, Aga F, Assebe Yadeta T et al. Self-care practices and correlates among patients with type 2 diabetes in Eastern Ethiopia: a hospital-based cross-sectional study. SAGE Open Med. 2022;10:20503121221107337. 35784669 10.1177/20503121221107337 PMC9244934

[44] Camara A, Baldé NM, Sobngwi-Tambekou J et al. Poor glycemic control in type 2 diabetes in the South of the Sahara: the issue of limited access to an HbA1c test. Diabetes Res Clin Pract. 2015;108(1):187–92. 25697633 10.1016/j.diabres.2014.08.025

[45] Tekalegn Y, Addissie A, Kebede T et al. Magnitude of glycemic control and its associated factors among patients with type 2 diabetes at Tikur Anbessa Specialized Hospital, Addis Ababa, Ethiopia. PLoS One. 2018;13(3):e0193442. 29505602 10.1371/journal.pone.0193442 PMC5837131

[46] Fasil A, Biadgo B, Abebe M. Glycemic control and diabetes complications among diabetes mellitus patients attending at University of Gondar Hospital, Northwest Ethiopia. Diabetes Metab Syndr Obes. 2018;12:75–83. 30613158 10.2147/DMSO.S185614 PMC6306061

[47] Eticha T, Mulu A, Gebretsadik H et al. Factors associated with poor glycemic control in type 2 diabetic patients investigated at Ayder Referral Hospital, Mekelle, Ethiopia. IJPPR. 2016;6(3):160–71.

[48] Chen S, Shen Y, Liu Y-H et al. Impact of glycemic control on the association of endothelial dysfunction and coronary artery disease in patients with type 2 diabetes mellitus. Cardiovasc Diabetol. 2021;20(1):64. 33714276 10.1186/s12933-021-01257-y PMC7956110

